# 真性红细胞增多症患者干扰素和（或）羟基脲治疗后症状负荷改善与血液学疗效之间的关系

**DOI:** 10.3760/cma.j.issn.0253-2727.2021.08.004

**Published:** 2021-08

**Authors:** 丹 刘, 泽锋 徐, 铁军 秦, 士强 曲, 秀娟 孙, 冰 李, 丽娟 潘, 志坚 肖

**Affiliations:** 中国医学科学院北京协和医学院血液病医院（中国医学科学院血液学研究所），实验血液学国家重点实验室，国家血液系统疾病临床医学研究中心，天津 300020 State Key Laboratory of Experimental Hematology, National Clinical Research Center for Blood Diseases, Institute of Hematology & Blood Diseases Hospital, Chinese Academy of Medical Sciences & Peking Union Medical College, Tianjin 300020, China

**Keywords:** 真性红细胞增多症, 骨髓增殖性肿瘤总症状评估量表, 血液学疗效, 干扰素, 羟基脲, Polycythemia vera, Myeloproliferative Neoplasm Symptom Assessment Form Total Symptom Score, Response, Interferon, Hydroxyurea

## Abstract

**目的:**

探讨真性红细胞增多症（PV）患者干扰素和（或）羟基脲治疗后症状负荷改善与血液学疗效之间的关系。

**方法:**

对190例符合WHO（2016）诊断分型标准、接受干扰素和（或）羟基脲连续治疗≥6个月的PV患者，分别应用外周血细胞计数和骨髓增殖性肿瘤总症状评估量表（MPN-10）评价患者血液学疗效和症状负荷。

**结果:**

全部190例PV患者中，男93例（48.9％），女97例（51.1％）。进行MPN-10评分时，患者中位年龄为60（32～82）岁。全部患者中位MPN-10总分为9（0～67）分，干扰素+羟基脲组（27例）为11（0～67）分，显著高于干扰素组［64例，6（0～56）分，*P*＝0.019］和羟基脲组［99例，9（0～64）分，*P*＝0.047］，而干扰素组与羟基脲组差异无统计学意义（*P*＝0.421）。28.9％（55/190）的患者存在重度症状（单项症状评分≥7分或总分≥44分），干扰素组、羟基脲组、干扰素+羟基脲组重度症状患者占比分别为23.4％、29.3％、40.7％，组间比较差异均无统计学意义（*P*>0.05）。全部190例患者中，进行MPN-10评分时71例（37.4％）获得完全血液学缓解（CHR），仅55例（28.9％）获得疾病充分控制（获得CHR且无重度症状）。未获得疾病充分控制的患者包括：血细胞增高80例（42.1％），获得CHR但伴重度症状16例（8.4％），血细胞增高且伴重度症状39例（20.5％）。PLT>400×10^9^/L患者中存在重度症状患者比例、MPN-10总分中位数均高于PLT≤400×10^9^/L患者［40.8％（20/49）对24.8％（35/141），*P*＝0.044；14（0～67）分对7（0～56）分，*P*＝0.038］。PLT>400×10^9^/L是存在重度症状的危险因素（*HR*＝2.089，95％*CI* 1.052～4.147，*P*＝0.035）。

**结论:**

经干扰素和（或）羟基脲治疗后，PV患者症状负荷未获满意控制仍较突出，部分患者虽然获得CHR，仍存在重度症状负荷。PLT>400×10^9^/L是干扰素和羟基脲治疗后患者存在重度症状的危险因素。

## Abstract (en)

**Objective:**

To explore the relationship between symptom burden and hematologic responses after treatment with interferon and/or hydroxyurea in patients with polycythemia vera (PV).

**Methods:**

Hematologic responses after continuous treatment with interferon and/or hydroxyurea for six months were evaluated in 190 patients with PV using the Myeloproliferative Neoplasm Symptom Assessment Form Total Symptom Score (MPN-10 score). In all patients, the PV diagnosis was based on the 2016 World Health Organization diagnostic definitions.

**Results:**

The study cohort comprised 93 (48.9％) male and 97 (51.1％) female patients. The median age at the time of MPN-10 assessment was 60 (32–82) years. The median MPN-10 score of the entire cohort was 9 (range, 0–67). The median MPN-10 score of patients treated with interferon plus hydroxyurea (*n*＝27) was 11 (0–67), which was significantly higher than those of patients treated with interferon only (*n*＝64) (6[0–56], *P*＝0.019) or hydroxyurea only (*n*＝99) (9[0–64], *P*＝0.047), whereas the median MPN-10 score was not significantly different between those treated with interferon only and hydroxyurea only (*P*＝0.421). The rate of severe symptom burden (i.e., any single symptom burden score ≥ 7 and/or total score ≥ 44) was 28.9％ (55/190) in the entire cohort, whereas the rate of severe symptom burden was not significantly different among the interferon only (23.4％), hydroxyurea only (29.3％), and interferon plus hydroxyurea (40.7％) groups (*P*>0.05 for all two-group comparisons). When evaluating MPN-10 score, 37.4％ (71/190) of the patients achieved complete hematologic remission (CHR). Only 28.9％ (55/190) patients had adequate disease control, defined as CHR without severe symptom burden. Reasons for inadequate disease control were evaluating blood counts alone, severe symptom burden alone, and evaluating blood counts accompanied with severe symptom burden in 42.1％ (80/190), 8.4％ (16/190), and 20.5％ (39/190) of the patients, respectively. Compared to the patients with a platelet count ≤ 400×10^9^/L, those with a platelet count > 400×10^9^/L had a significantly higher rate of severe symptom burden (40.8％ [20/49] *vs* 24.8％ [35/141], *P*＝0.044) and a higher median MPN-10 score (14[0–67] *vs* 7[0–56], *P*＝0.038). Platelet count > 400×10^9^/L was associated with an increased risk of severe symptom burden (hazard ratio, 2.089; 95％ confidence interval, 1.052–4.147, *P*＝0.035).

**Conclusion:**

Symptoms related to disease after treatment with interferon and/or hydroxyurea were rather universal in patients with PV. Some patients still experienced severe symptom burden despite achieving CHR. Platelet count > 400 × 10^9^/L was associated with an increased risk of severe symptom burden in patients with PV treated with interferon and/or hydroxyurea.

真性红细胞增多症（PV）是一种获得性骨髓增殖性肿瘤（MPN），其特点为造血干细胞克隆性增殖，导致骨髓红系、粒系、巨核系三系过度增生，95％以上的PV患者有JAK2基因突变（V617F或外显子12）[1]–[3]。既往PV的疗效判定标准主要关注临床血液学及骨髓组织病理学两方面[4]。本中心以往研究显示体质性症状是导致这些患者的情绪负担沉重、工作效率降低及经济困难的主要原因[5]。为了探讨PV患者治疗后症状负荷和血液学疗效之间的关系，我们同时采用MPN患者症状负荷评估量表（MPN-10）和外周血细胞计数对190例接受人重组干扰素α2b（简称干扰素）和（或）羟基脲治疗的患者进行疗效评估，现报道如下。

## 病例与方法

1. 病例：本研究纳入2012年1月至2020年10月在中国医学科学院血液病医院就诊的190例PV患者。纳入标准：①诊断符合WHO（2016）分型标准[6]；②评估症状负荷前，干扰素和（或）羟基脲连续治疗≥6个月；③有评估症状负荷前3个月内外周血细胞计数血液学疗效评估结果；④年龄≥18岁，意识清楚，无精神疾病、认知障碍，能够准确表达意见，愿意配合本研究。

2. 症状负荷评估：症状负荷是指患者依据MPN-10量表[7]对填表前1周内自我感觉症状进行单项和所有项目自我评估的得分值。采用以下方式完成MPN-10量表评估：①门诊随诊患者采用面对面形式（62例）；②通过微信（54例）或电话（74例）。单项症状评分0分为无症状，1～3分为轻度症状，4～6分为中度症状，≥7分为重度症状。单项症状评分≥7分和（或）总分≥44分定义为存在重度症状负荷。

3. 治疗方案：190例患者治疗方案包括：①64例（33.7％）接受干扰素治疗；②99例（52.1％）接受羟基脲治疗；③27例（14.2％）接受干扰素+羟基脲治疗。干扰素起始剂量为300万单位（IU），每周3次；羟基脲起始剂量为20 mg·kg^−1^·d^−1^。干扰素及羟基脲剂量由医师根据患者外周血细胞计数情况进行调整。

4. 疗效判断标准：血液学疗效判断标准依据欧洲白血病联盟（ELN）及MPN国际工作组（IWG-MRT）制定的疗效判断标准[8]，完全血液学缓解（CHR）是指红细胞压积（HCT）<45％、WBC<10×10^9^/L、PLT≤400×10^9^/L。一系或多系未达到CHR标准定义为血细胞增高。达到CHR并且无重度症状定义为疾病充分控制。MPN-10评分距外周血细胞计数间隔时间中位数为0（0～3）个月，间隔时间≤1、2、3个月的患者分别占85.8％（163/190）、5.3％（10/190）、8.9％（17/190）。

5. 染色体核型分析及二代测序检测：染色体核型分析方法见文献[9]，二代测序方法见文献[10]。

6. 统计学处理：偏态分布计量资料以中位数表示，组间比较采用非参数Mann-Whitney *U*检验。率的比较采用Fisher确切概率法。存在重度症状影响因素分析采用Logistic回归模型分析。随访时间定义为从确诊日期至最后随访日期或死亡日期。所有数据采用SPSS 26.0软件包进行统计学处理。

## 结果

1. 临床特征：全部190例PV患者中，男93例（48.9％），女97例（51.1％），MPN-10评分时的中位年龄为60（32～82）岁。MPN-10评分距确诊PV的中位时间为59.5（12～238）个月。MPN-10评分时血常规（中位数）：HGB 148（107～208）g/L，HCT 44.3％（31.3％～65.3％），WBC 7.79（2.84～30.38）×10^9^/L，PLT 297（99～954）×10^9^/L。30.1％（56/186）的患者在MPN-10评分前有血栓史。1.6％（2/92）的患者确诊时检出克隆性染色体核型异常，其中1例为“47, XX, +9[15]”，另1例为“46, XY, del（15）（?q24）, add（17）（p13）[20]”。JAK2V617F突变阳性183例（96.3％），JAK2外显子12突变阳性1例（0.5％）。21例进行了二代测序基因突变检测，9例（42％）患者检出JAK2基因外的其他突变基因，其中NOTCH1基因突变3例，TET2基因突变2例。

全部190例患者的中位随访时间为75（12～252）个月，MPN-10评分距治疗开始的中位时间为52（8～238）个月；干扰素组、羟基脲组、干扰素+羟基脲组的中位随访时间分别为74（17～231）、72（12～237）、98（17～252）个月；干扰素组、羟基脲组MPN-10评分距治疗开始的中位时间分别为50（11～135）、55（8～157）个月；干扰素+羟基脲组距干扰素、羟基脲治疗开始的中位时间分别为34（1～120）、55（14～238）个月。随访期间17例（9.1％）患者发生血栓事件，1例（0.5％）患者死亡，1例（0.5％）患者转为PV后骨髓纤维化（post-PV-MF），1例（0.5％）患者转为急性髓系白血病（AML）。

在门诊面对面进行MPN-10评分的患者中，接受干扰素、干扰素+羟基脲、羟基脲治疗的患者比例分别为40.3％（25/62）、29.0％（18/62）、30.6％（19/62）；在微信评分的54例患者中，接受干扰素、干扰素+羟基脲、羟基脲治疗的患者比例分别为38.9％（21/54）、11.1％（6/54）、50.0％（27/54）；在电话评分的74例患者中，接受干扰素、干扰素+羟基脲、羟基脲治疗的患者比例分别为24.3％（18/74）、4.1％（3/74）、71.6％（53/74）。

2. 症状负荷评估：全部190例患者的MPN-10总分中位数为9（0～67）分。最常见的症状为疲劳（65.2％）、皮肤瘙痒（49.4％）和活动力不佳（41.6％）（图1）。55例（28.9％）患者存在重度症状，其中26例（13.7％）存在1种重度症状，14例（7.4％）存在2种重度症状，15例（7.9％）存在≥3种重度症状。9例（4.7％）患者症状负荷总分≥44分。

面对面和电话方式进行MPN-10评分的患者中，存在重度症状患者占比差异没有统计学意义［35.％（22/62）对32.4％（24/74），*P*＝0.708］，微信评分患者中存在重度症状患者占比（9例，16.7％）显著低于面对面和电话评估的患者（*P*＝0.022，*P*＝0.044）。

干扰素组64例患者中，最常见的症状为疲劳（68.8％）和皮肤瘙痒（45.3％）。15例（23.4％）患者存在重度症状，其中6例（9.4％）存在1种重度症状，5例（7.8％）存在2种重度症状，4例（6.2％）存在≥ 3种重度症状。4例（6.2％）患者症状负荷总分≥44分。

羟基脲组99例患者中，最常见的症状为疲劳（60.6％）、皮肤瘙痒（54.6％）、活动力不佳（44.5％）和注意力不集中（44.5％）。29例（29.3％）患者存在重度症状，其中16例（16.2％）存在1种重度症状，6例（6.1％）存在2种重度症状，7例（7.0％）存在≥3种重度症状。4例（4.0％）患者症状负荷总分≥44分。

干扰素+羟基脲组的27例患者中，最常见的症状为疲劳（60.6％）、活动力不佳（51.8％）和骨痛（51.8％）。11例（40.7％）患者存在重度症状，其中4例（14.8％）存在1种重度症状，3例（11.1％）存在2种重度症状，4例（14.8％）存在≥3种重度症状。2例（7.4％）患者症状负荷总分≥44分。

干扰素+羟基脲组MPN-10总分中位数为11（0～67）分，显著高于干扰素组［6（0～56）分，*z*＝−2.338，*P*＝0.019］和羟基脲组［9（0～64）分，*z*＝−1.983，*P*＝0.047］，而干扰素组与羟基脲组总分中位数差异无统计学意义（*z*＝−0.805，*P*＝0.421）。干扰素组与羟基脲组（23.4％对29.3％，*P*＝0.472）、干扰素组与干扰素+羟基脲组（23.4％对40.7％，*P*＝0.128）、羟基脲组与干扰素+羟基脲组（29.3％对40.7％，*P*＝0.351）存在重度症状患者比例差异均无统计学意义。干扰素+羟基脲组存在重度活动力不佳的患者占比为25.9％（7/27），高于羟基脲组［6.1％（6/99），*P*＝0.007］和干扰素组［3.1％（2/64），*P*＝0.002］。

与羟基脲组比较，在应用干扰素的患者（干扰素组及干扰素+羟基脲组）中，骨痛［37.4％（34/91）对15.2％（15/99），*P*＝0.001］、发热［14.3％（13/91）对3.0％（3/99），*P*＝0.007］、体重减轻［29.7％（27/91）对15.2％（15/99），*P*＝0.012］的患者占比更高（图1）。

**图1:**
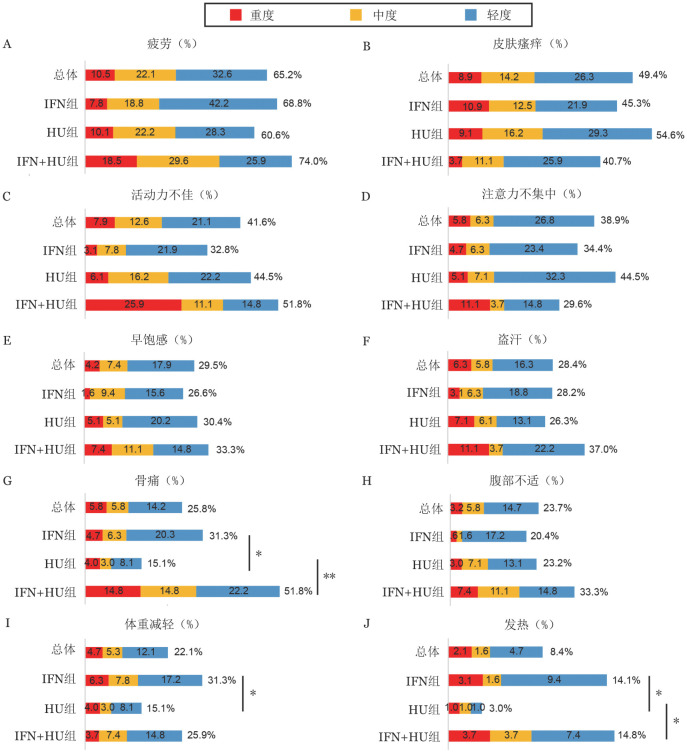
真性红细胞增多症患者干扰素（IFN）和（或）羟基脲（HU）治疗后症状负荷（*P<0.05，**P<0.01）

3. 血液学疗效评估：全部190例PV患者中，评估症状负荷时71例（37.4％）获得CHR，外周血细胞计数1系增高77例（40.5％），2系增高24例（12.6％），3系增高18例（9.5％）。WBC≥10×10^9^/L 42例（22.1％），HCT≥45％ 88例（46.3％），PLT>400×10^9^/L 49例（25.8％）。干扰素组获得CHR患者比例高于羟基脲组［53.1％（34/64）对26.3％（26/99），*P*＝0.001］。干扰素+羟基脲组40.7％（11/27）获得CHR，与干扰素组、羟基脲组比较差异无统计学意义（*P*＝0.360、*P*＝0.158）。

4. 患者症状负荷与外周血血细胞计数疗效之间的关系：全部190例患者中，获得疾病充分控制（取得CHR且无重度症状）的患者仅占28.9％（55/190），71.1％（135/190）患者疾病未获得充分控制，包括：42.1％（80/190）患者血细胞增高不伴有重度症状，8.4％（16/190）患者获得CHR但伴有重度症状，20.5％（39/190）患者血细胞增高且伴有重度症状。

干扰素组疾病充分控制患者占比为42.2％（27/64），34.4％（22/64）的患者血细胞增高不伴有重度症状，10.9％（7/64）的患者获得CHR但伴有重度症状，12.5％（8/64）的患者血细胞增高且伴有重度症状。

羟基脲组疾病充分控制患者占比为20.2％（20/99），50.5％（50/99）患者血细胞增高不伴有重度症状，6.1％（6/99）患者获得CHR但有伴重度症状，23.3％（23/99）患者血细胞增高并且伴有重度症状。

干扰素+羟基脲组疾病充分控制患者占比为29.6％（8/27），29.6％（8/27）的患者血细胞增高不伴有重度症状，11.1％（3/27）的患者获得CHR但伴有重度症状患者，29.6％（8/27）的患者血细胞增高且伴有重度症状。

干扰素组疾病充分控制的患者比例显著高于羟基脲组（42.2％对20.2％，*P*＝0.004）；干扰素组与干扰素+羟基脲组（42.2％对29.6％，*P*＝0.347）、羟基脲组与干扰素+羟基脲组（20.2％对29.6％，*P*＝0.305）比较，差异无统计学意义。

22.5％（16/71）的获得CHR患者存在重度症状，其中8例（11.％）存在1种重度症状，5例（7.0％）存在2种重度症状，3例（4.2％）存在≥3种重度症状。在未获得CHR患者中，存在重度症状的患者占32.8％（39/119），其中存在1、2、≥3种重度症状的患者分别为18例（15.1％）、9例（7.6％）、12例（10.1％）。获得CHR、未获得CHR患者存在重度症状的患者占比分别为22.5％、32.8％（*P*＝0.141）、MPN-10总分中位数分别为8（0～56）分、9（0～67）分（*z*＝−1.141，*P*＝0.254）（图2）。

**图2:**
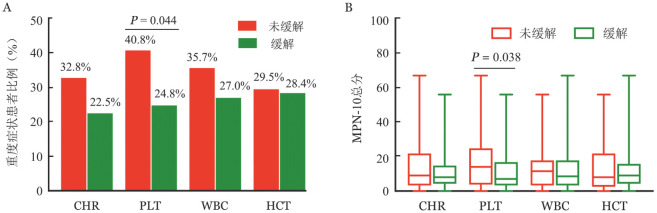
真性红细胞增多症患者血液学分组患者的症状负荷 CHR：完全血液学缓解；HCT：红细胞压积；A：血液学疗效分组患者存在重度症状患者比例的比较；B：血液学疗效分组患者MPN-10总分的比较

PLT>400×10^9^/L组存在重度症状患者比例、MPN-10总分均高于PLT≤400×10^9^/L组［40.8％（20/49）对24.8％（35/141），*P*＝0.044；14（0～67）分对7（0～56）分，*z*＝−2.080，*P*＝0.038］（图2）。进一步分析提示，PLT>400 × 10^9^/L组存在重度早饱感［12.2％（9/149）对1.4％（2/141），*P*＝0.004］、重度腹部不适［8.2％（4/49）对1.4％（2/141），*P*＝0.039］、重度皮肤瘙痒［18.4％（9/49）对5.7％（8/141），*P*＝0.016］的患者比例均高于PLT≤400 × 10^9^/L组。PLT>400×10^9^/L是存在重度症状的危险因素（*HR*＝2.089，95％ *CI* 1.052～4.147，*P*＝0.035），HCT≥45％、WBC≥10×10^9^/L以及治疗方案对存在重度症状的没有显著影响（表1）。

**表1:** 真性红细胞增多症患者干扰素和（或）羟基脲治疗后重度症状影响因素分析

影响因素	*HR*	95％*CI*	*P*值
年龄≥60岁	1.590	0.844～2.996	0.151
男性	0.601	0.318～1.136	0.117
血栓史	0.933	0.468～1.862	0.845
获得CHR	0.597	0.304～1.173	0.134
WBC≥10×10^9^/L	1.500	0.724～3.106	0.275
HCT≥45％	1.056	0.563～1.979	0.866
PLT>400×10^9^/L	2.089	1.052～4.147	0.035
干扰素治疗	0.658	0.330～1.311	0.234
羟基脲治疗	1.036	0.553～1.940	0.913
干扰素+羟基脲治疗	1.859	0.801～4.315	0.149

注：CHR：血液学缓解；HCT：红细胞压积

## 讨论

干扰素与羟基脲对MPN患者发挥治疗作用的机制存在差异。羟基脲是一种核糖核苷酸还原酶抑制剂，可触发双链DNA在复制叉附近断裂，从而抑制细胞核及线粒体内DNA复制，导致细胞及线粒体功能障碍[11]。此外，研究发现羟基脲可通过诱导外周血间充质干细胞衰老，通过旁观者效应抑制JAK2V617F阳性人红白血病细胞的增殖[11]。研究发现TNF-α表达增高可促进MPN恶性克隆增殖，PV及原发性血小板增多症（ET）患者经聚乙二醇干扰素治疗后，其造血祖细胞TNF-α表达减低，从而抑制MPN恶性克隆增殖，使造血细胞多克隆性得到修复，但经羟基脲治疗后的患者造血祖细胞TNF-α表达水平没有发生改变[12]。近期一项单细胞测序研究发现，经干扰素α治疗后的ET患者，其JAK2V617F纯合突变的造血干细胞重新进入细胞周期静止期，而JAK2V617F杂合突变的造血干细胞凋亡作用增强，提示进入静止期的细胞是疾病复发的来源[13]。干扰素对于造血干祖细胞的作用是其发挥分子学和血液学疗效的基础。

伴JAK2基因突变MPN患者的JAK-STAT信号通路异常激活，不仅造成骨髓细胞异常增殖，也伴随细胞因子分泌异常[14]。而细胞因子水平改变与MPN相关疾病症状（盗汗、骨痛、体重减轻等）相关[15]。约40％的PV患者伴脾大[16]，脾大与早饱感、腹部不适等症状相关。

本研究发现，经过半年及以上干扰素和（或）羟基脲治疗，PV患者疾病相关症状仍普遍存在。部分患者虽获得CHR，仍存在重度症状。这可能与干扰素、羟基脲对于疾病相关细胞因子异常、脾大等作用有限有关，也可能与干扰素可引起流感样症状、发热、骨痛等并发症相关。疲劳是干扰素和（或）羟基脲治疗后PV患者最常见症状，存在于近70％的患者，这与既往研究结果一致[7],[17]。28.9％患者存在重度症状，联合外周血细胞计数，仅28.9％的的患者疾病得到充分控制。干扰素组CHR率最高，既往研究也提示干扰素治疗PV患者可获得较好的血液学缓解和分子学缓解[18]–[19]，但患者疾病相关症状并未明显改善，甚至干扰素相关不良反应，如发热、骨痛等症状更为突出。干扰素+羟基脲联合治疗的患者，虽然也取得了较好的血液学疗效，但这部分患者具有比干扰素组或羟基脲组更重的症状负荷。因此，干扰素和（或）羟基脲治疗PV患者，疾病得到充分控制的患者比例不高，部分存在重度症状负荷的患者，应调整治疗策略。

既往两个多中心Ⅲ期随机对照临床试验提示，PV患者可获取的最佳治疗（best available treatment, BAT）（包括羟基脲、干扰素、阿那格雷等）均不能改善患者疾病相关症状负荷；而Janus激酶（JAK）抑制剂芦可替尼可在短期内（4周）显著改善PV患者疾病相关症状负荷，治疗28～32周时，约50％的患者症状负荷总分至少下降50％[20]–[21]。此外，一项前瞻性临床研究比较羟基脲、聚乙二醇干扰素和芦可替尼治疗MPN患者的疗效，该研究纳入了PV、原发性血小板增多症（ET）和骨髓纤维化（PMF）患者，结果也提示患者在羟基脲或聚乙二醇干扰素治疗3～24个月MPN-10总分均没有显著变化，而芦可替尼组从第3个月开始MPN-10评分显著低于羟基脲或聚乙二醇干扰素组[22]。除了显著减轻症状负荷，芦可替尼治疗的PV患者获得CHR、HCT<45％的患者比例也显著高于BAT患者[20]–[21]。目前，欧盟和美国已批准芦可替尼用于治疗羟基脲耐药、不耐受及治疗反应不佳的PV患者。

本研究提示，PV患者干扰素和（或）羟基脲治疗后是否获得CHR、是否获得WBC或HCT缓解，对患者MPN-10总分和存在重度症状患者比例无显著影响，而PLT>400×10^9^/L是存在重度症状的危险因素。Grunwald等[23]研究发现是否获得CHR、是否获得PLT或HCT缓解的PV患者MPN-10总分、重度症状患者比例无显著差异，但WBC>10×10^9^/L的患者MPN-10总分平均分高于WBC 10×10^9^/L患者（20.2对18.0，*P*＝0.036）。本研究纳入患者均为干扰素和（或）羟基脲治疗，而Grunwald等[23]研究中仅约50％患者应用干扰素或羟基脲治疗，这可能是与本研究产生差异的原因。血小板可分泌炎性因子，如趋化因子CXCL4、CCL5、CXCL12、CXCL16等，引起局部炎症和血管栓塞[24]，这可能是PLT增高患者症状负荷更重的原因。这也提示干扰素和（或）羟基脲治疗的PV患者通过控制PLT水平，可能会减轻患者疾病相关症状负荷。

本研究结果显示，PV患者经干扰素和（或）羟基脲治疗后，仅少数患者疾病得到充分控制，大部分患者疾病相关症状仍普遍存在。对于干扰素和（或）羟基脲治疗效果欠佳的患者，尤其是存在重度症状负荷的患者，可考虑调整为芦可替尼治疗。此外，干扰素和（或）羟基脲治疗的患者，通过控制PLT≤400×10^9^/L，可能会减轻患者症状负荷，但这还需要大系列临床研究加以验证。本研究的主要不足是患者自填量表的完成方式不一致，有可能给结果带来一定的偏倚。
